# Substrate-selective COX-2 inhibition by IMMA attenuates posttraumatic headache via endocannabinoid modulation and neuroinflammatory suppression

**DOI:** 10.1186/s10194-025-02116-x

**Published:** 2025-08-12

**Authors:** Jie Wen, Mikiei Tanaka, Yumin Zhang

**Affiliations:** Department of Anatomy, Physiology and Genetics, Uniformed Services University of the Health Sciences, 4301 Jones Bridge Road, Bethesda, MD 20814 USA

## Abstract

**Background:**

Posttraumatic headache (PTH) is a common and debilitating consequence of traumatic brain injury (TBI), characterized by neuroinflammation and pain hypersensitivity. Current treatments are limited, and novel therapeutics are needed. Indomethacin morpholinamide (IMMA), a substrate-selective cyclooxygenase-2 (COX-2) inhibitor, enhances endocannabinoid signaling without disrupting prostaglandin homeostasis and may offer a mechanistically distinct approach to managing PTH.

**Methods:**

Male C57BL/6J mice were subjected to repetitive mild TBI (rmTBI) using the Closed-Head Impact Model of Engineered Rotational Acceleration (CHIMERA) and treated with IMMA (10 mg/kg, i.p.) daily for 7 days post-injury. Mechanical allodynia was assessed using von Frey stimulation of the periorbital region. Neuroinflammation was evaluated through immunohistochemistry in the trigeminal ganglion (TG) and trigeminal nucleus caudalis (TNC). Endocannabinoid and prostaglandin levels were quantified by mass spectrometry and enzyme immunoassay, respectively.

**Results:**

IMMA significantly reduced rmTBI-induced periorbital allodynia, microglial and astrocyte activation, and CGRP expression in the TG and TNC. It also preserved meningeal mast cell integrity and elevated cortical anandamide (AEA) levels without altering prostaglandin E₂ (PGE₂) production, supporting a mechanism that enhances cannabinoid signaling while sparing COX-2-mediated prostaglandin synthesis.

**Conclusion:**

IMMA effectively attenuates neuroinflammation and pain hypersensitivity in the acute phase of PTH through a distinct mechanism that preserves endocannabinoid tone without suppressing physiological prostaglandins. While these results highlight its promise as a novel therapeutic strategy, further studies are warranted to determine its efficacy during the chronic phase of PTH and across anatomically targeted regions.

**Supplementary Information:**

The online version contains supplementary material available at 10.1186/s10194-025-02116-x.

## Background

Traumatic brain injury (TBI) remains a significant public health challenge, frequently leading to posttraumatic headache (PTH), one of the most common and debilitating consequences. PTH resembles migraine-like or tension type headache phenotypes, significantly impacting patients’ quality of life [1–3]. The pathophysiology of PTH is complex, involving both peripheral and central mechanisms. Mechanical and biochemical insults following TBI activate meningeal nociceptors, initiate inflammatory cascades, and stimulate the release of prostaglandins, cytokines, and calcitonin gene-related peptide (CGRP) within the trigeminal pain pathway [4–6]. These events collectively drive neuronal sensitization and amplify pain perception, contributing to the development and persistence of headache symptoms.

Among the mediators implicated in this process, CGRP, a neurogenic peptide expressed in the trigeminal neurons, plays a crucial role in migraine and PTH by promoting vasodilation, inflammation and nociceptive sensitization [7, 8]. The increase of CGRP levels is associated with headache severity, and both monoclonal antibodies against CGRP and the CGRP receptor antagonists have shown efficacy in migraine treatment [9–11]. However, given the CGRP’s regulatory roles in vascular and immune functions, chronic inhibition of its signaling may pose potential cardiovascular risks [12, 13]. Additionally, delayed administration of anti-CGRP antibodies is less effective in the PTH animal models [5, 14], suggesting the involvement of CGRP independent mechanisms.

The endocannabinoid system (ECS) represents a key regulatory pathway involved in pain and inflammation. ECS consists of endogenous cannabinoids, primarily anandamide (AEA) and 2-arachidonoylglycerol (2-AG), which activate the cannabinoid type 1 (CB1) and type 2 (CB2) receptors [15, 16]. CB1 receptors, primarily located in the central nervous system, modulate nociceptive transmission by inhibiting the release of excitatory neurotransmitters, while CB2 receptors, predominantly expressed in peripheral immune cells, reduce inflammation by limiting cytokine production and mast cell activity [17–20]. For the degradation of endocannabinoids, AEA is primarily hydrolyzed by fatty acid amide hydrolase (FAAH), while 2-AG is metabolized by monoacylglycerol lipase (MAGL). Enhancement of ECS signaling through FAAH or MAGL inhibition has been shown to reduce pain in various preclinical models. In the formalin-induced inflammatory pain model, selective inhibition of MAGL with JZL184 increased 2-AG levels in mouse hind paw tissue and attenuated pain behavior, and the analgesic effect was reversed by either CB1 or CB2 receptor antagonist [21]. FAAH knock out mice were shown to have analgesic phenotype, exhibiting reduced pain sensation in the hot plate test, the formalin test and the tail flick test [22]. Using a repetitive mild TBI (rmTBI) mouse model induced by the closed head impact model of engineered rotational acceleration (CHIMERA), we found that the selective MAGL inhibitor MJN110 attenuated cephalic allodynia by enhancing 2-AG signaling, further supporting the involvement of endocannabinoids in post-TBI pain modulation [4].

Besides hydrolysis, endocannabinoids are subject to oxygenation for degradation. COX-2 but not COX-1 converts AEA and 2-AG into pro-inflammatory prostaglandin ethanolamides (PG-EAs) and prostaglandin glycerol esters (PG-Gs), thereby reducing the endocannabinoid availability and counteracting their analgesic effects [23–26]. It has been reported that some enantiomers of nonsteroidal anti-inflammatory drugs (NSAIDs) can selectively inhibit endocannabinoid oxygenation, but not arachidonic acid (AA) metabolism. Therefore, these substrate-selective COX-2 inhibitors (SSCIs) are able to boost endocannabinoid signaling, while maintaining homeostatic prostaglandin function [27]. Administration of indomethacin morpholinamide (IMMA), a well-characterized SSCI, was shown to reduce anxiety-like behavior by augmentation of brain levels of AEA and 2-AG without affecting non-endocannabinoid lipids and prostaglandin synthesis [28]. We have also demonstrated that IMMA effectively attenuates hyperalgesia and suppresses neuroinflammation by preserving endogenous cannabinoid signaling in a neuropathic pain mouse model [29]. Moreover, co-administration of IMMA with a low dose of FAAH inhibitor produces greater efficacy in alleviating neuropathic pain [30].

Microglia and astrocytes, the major glial cells in the central nervous system, become activated in response to rmTBI and contribute to neuroinflammation and pain amplification, particularly within the trigeminal nucleus caudalis (TNC), a key relay center for craniofacial nociceptive transmission [31, 32]. Meningeal mast cells are among the first immune responders to injury and release pro-inflammatory mediators to sensitize trigeminal afferents and exacerbate headache-like behaviors [33, 34]. Prostaglandin E_2_ (PGE_2_), synthesized from PGH_2_ by prostaglandin E synthases such as Pges1 and Pges2, plays a central role in inflammatory pain and central sensitization [35]. These molecules are upregulated in glia and immune cells after injury, and their modulation may play a critical role in regulating the development and persistence of PTH.

Building upon these findings, we hypothesized that IMMA could preserve endocannabinoid signaling and mitigate neuroinflammatory response. By targeting both COX and ECS pathways, IMMA treatment may offer a promising approach for the management of PTH.

##  Materials and methods

### Materials

IMMA, deuterated AEA (AEA-d4) and 2-AG (2-AG-d5) were purchased from Cayman Chemicals (Ann Arbor, MI). All other chemicals and reagents were purchased from Sigma (St. Louis, MO), unless stated otherwise.

### Animals

10-week-old, male C57BL/6J mice were obtained from the Jackson Laboratory (Bar Harbor, ME). Mice were group housed in temperature- and humidity- controlled vivarium cages containing cellulose-based bedding and a plastic enrichment device. A 14/10-hour light/dark cycle was maintained (lights on at 05:00, off at 19:00). Food and water were available *ad libitum*. A total of 138 animals were used in this study, and all experiments were conducted during the light phase. Animal care and experimental procedures were carried out in accordance with NIH guidelines and approved by the Uniformed Services University Animal Care and Use Committee (IACUC), and the Animal Care and Use Review Office (ACUCO) from the United States Department of Defense (DoD).

### Induction of repetitive mTBI

To induce mild traumatic brain injury (mTBI), we employed the Closed-Head Impact Model of Engineered Rotational Acceleration (CHIMERA), a well-characterized model system for replicating diffuse, non-penetrating brain injury in rodents [36, 37]. Mice were anesthetized using isoflurane (3% for induction, 2% for maintenance) and then secured in a supine position at an approximate 32° angle on the CHIMERA device. The head of each mouse remained unrestrained, with the frontal and parietal bones positioned flat over the opening in the head plate. A controlled impact delivering 0.7 joules of energy was administered to the bregma once daily over four consecutive days. This injury paradigm produced a mild TBI without evidence of skull fracture, gross anatomical disruption, histopathological brain damage, or prolonged loss of consciousness. Sham animals were subjected to identical handling and anesthesia protocols but did not receive the impact.

### Cephalic cutaneous allodynia

Mechanical allodynia was evaluated by recording withdrawal responses to tactile stimulation with von Frey filaments, as we previously described [4, 38]. Thresholds were determined using the “Up-Down” method [39] by an experimenter blinded to group allocation. Stimulation was delivered to the midline periorbital area, located above the eyes and associated with the ophthalmic branch of the trigeminal nerve. Hairs in this area were left intact to avoid irritation or sensitization from shaving. Mice were placed in tube restrainers and acclimated for at least 5 min daily over two days prior to testing. A set of von Frey filaments (2.44 to 4.31; 0.16–6.0 g) was applied perpendicularly until slight bending occurred. A positive response was defined as face stroking, head withdrawal, or head shaking after three consecutive stimuli, a criterion used to improve consistency in the facial area, which is sensitive to movement [40]. Adequate inter-stimulus intervals were used to minimize sensitization.

### Drug treatment

IMMA was prepared in a DMSO-cremophor-saline solution (1:1:18 ratio), which served as the vehicle control. Animals were randomly assigned into sham, TBI/vehicle, and TBI/IMMA treatment groups. IMMA was administered intraperitoneally (i.p.) 1 h after each impact, and continuing daily for an additional 3 days, completing a total treatment duration of 7 days.

### Immunohistochemistry

After euthanasia using a ketamine/xylazine cocktail (90 mg ketamine/10 mg xylazine per ml, administered at 10 µl/g body weight, i.p.), animals underwent intracardiac perfusion with ice-cold 1x PBS followed by 4% paraformaldehyde (PFA) in the same buffer. The trigeminal ganglion (TG) and brain were carefully dissected and stored in 4% PFA at 4 °C overnight. The tissues were then rinsed twice with 1x PBS and transferred to a 20% sucrose solution in PBS at 4 °C until they sank completely. Once settled, the tissues were embedded in Tissue Tek OCT and preserved at − 80 °C. Transverse sections of the brainstem and TG were cut at 25 μm thickness using Cryostat (Leica CM1950, Bannockburn, IL) and mounted onto Superfrost Plus slides for immunohistochemical analysis. Primary antibodies used for immunohistochemistry (IHC) included anti-rat Iba1 (1:300; Novus Biologicals, LLC, Centennial, CO), anti-mouse GFAP (1:500; Cell Signaling, Danvers, MA), and anti-mouse CGRP (1:100; Santa Cruz, CA). The staining process began with two PBS washes, followed by a 30-minute incubation at room temperature in a PBS blocking buffer containing 5% donkey serum and 0.3% Triton X-100. Primary antibodies were then applied in the same buffer and incubated overnight. On the following day, slides were washed three times with PBS containing 0.2% Triton X-100 and incubated for 1 h with secondary antibodies conjugated to Alexa Fluor 488, Alexa Fluor 594, or Alexa Fluor 655 (donkey anti-rabbit, mouse, or rat, 1:750; Thermo Fisher Scientific, Waltham, MA). After additional PBS washes, slides were cover slipped with Fluoroshield mounting medium containing DAPI. Fluorescence images were acquired using a Nikon Eclipse TE-2000 U microscope with identical settings applied across all samples. For analysis, images were converted to 8-bit grayscale and processed in ImageJ (NIH) using consistent thresholding and particle analysis parameters. The image scale was calibrated using the microscope’s metadata, and pixel-to-micrometer conversions were uniformly applied. Immunoreactive cells for Iba1, GFAP, and CGRP were quantified from 5 to 7 serial coronal sections of the TG or TNC per animal. Data were expressed as the average number of cells or fluorescence intensity per mm².

For TNC analysis, the region was identified using anatomical landmarks corresponding to − 7.5 to − 8.5 mm from bregma, guided by Paxinos and Franklin’s Mouse Brain Atlas. Immunoreactivity was assessed bilaterally from serial coronal sections within this range. Regions of interest (ROIs) were manually defined using DAPI counterstaining and structural contours under low magnification to ensure reproducibility. All image analyses were performed by investigators blinded to group allocation. Negative controls were included by omitting primary antibodies.

### Mast cell density and degranulation analysis

Animals were deeply anesthetized with ketamine/xylazine and transcardially perfused with 100 mL of PBS, followed by 100 mL of 4% PFA. The skull cap was then removed and immersed in 4% PFA at 4 °C overnight. The following day, the PFA was replaced with 20% sucrose in PBS, and the tissue was stored at 4 °C until further processing. For mast cell analysis, the dura mater was carefully dissected from the frontonasal suture to the coronal suture and mounted onto a glass slide. After complete drying, the tissue was stained with Toluidine Blue suspension (VitroVivo Biotech, Gaithersburg, MD) for 3 min at room temperature. Slides were then rinsed three times with distilled water, dehydrated through a graded ethanol series, and cleared in xylene before cover slipping, according to the manufacturer’s protocol.

Intact mast cells were identified by their dense dark blue staining, indicating preserved secretory granules. In contrast, degranulated mast cells were defined by dispersed granules or a loss of staining around the cell. Total and degranulated mast cells were quantified in four consecutive visual views adjacent to the sagittal sinus (Bregma + 0.5 mm to + 1.5 mm).

### qRT-PCR

On the seventh day following rmTBI, mice were euthanized, and their entire brains and trigeminal ganglia were swiftly removed and flash-frozen. For the TNC, the frozen brain was sectioned coronally into 200 μm slices, spanning from − 8.2 mm to −6.8 mm relative to bregma, using Cryostat. The TNC was then excised from these frozen sections. The tissues were homogenized in 0.5 ml of TRIzol, and total RNA was extracted using a hybrid approach combining TRIzol phase separation with spin column purification, including on-column DNase I digestion (ZymoResearch). Complementary DNA was synthesized using the MAXIMA cDNA synthesis kit, followed by quantitative real-time PCR (qRT-PCR) with SYBR Green PowerUp Master Mix (Thermo Fisher Scientific) to evaluate the relative expression of the target genes, normalized to GAPDH. Relative gene expression levels were calculated using the 2^−ΔΔCt^ method.

### Mass spectrometry

The brain tissue was homogenized in a mixture of 40 µl of 0.02% trifluoroacetic acid and 60 µl of acetonitrile, spiked with internal standards 2-AG-d5 (40 ng) and AEA-d4 (20 pg) (Cayman Chemicals, Ann Arbor, MI, USA), using a Potter homogenizer at 4 °C. The resulting homogenate was fully dissolved in 1 ml of acetonitrile with vortexing and incubated at 4 °C overnight. The homogenate was then centrifuged at 1,000 g for 10 min to pellet debris. The supernatant was collected and dried under a stream of nitrogen gas in a water bath maintained at approximately 35 °C. The extracted lipids were resuspended in 100 µl of acetonitrile and stored at − 80 °C until analysis. Levels of 2-AG and AEA were quantified using liquid chromatography coupled with tandem mass spectrometry (LC-MS/MS), following our previously established protocol [41–43].

### Enzyme linked immunosorbent assay (ELISA)

For PGE_2_ assay, mouse TG tissue at day 7 post-injury was homogenized with 40 µL of 0.02% trifluoroacetic acid and 100 µL of acetonitrile on ice. The homogenate was then further dispersed in 1 mL of acetonitrile by vortex and left at 4 °C overnight. On the next day the supernatant after centrifugation at 2000 g for 5 min to remove the debris was evaporated, then reconstituted with enzyme immunoassay buffer supplemented in the PGE_2_ ELISA kit (Cayman Chemical, Ann Arbor, MI, USA). ELISA was performed following the manufacturer’s protocol.

### Statistical analysis

Statistical analyses were conducted using GraphPad Prism 9 and R (v4.2.2). For comparisons involving three experimental groups, one-way analysis of variance (ANOVA) was utilized, followed by Tukey’s or Bonferroni post hoc tests as appropriate. Repeated measures ANOVA was applied to analyze behavioral data collected over multiple time points. Statistical outliers were identified by the Extreme Studentized Deviate method using GraphPad Prism 9. Statistical significance was set at *p* < 0.05. Data are presented as the mean ± standard error of the mean (SEM). Sample sizes were determined based on power analyses and prior studies to ensure adequate statistical power.

## Results

### IMMA suppresses periorbital allodynia in repetitive mTBI mice

**Fig. 1:**
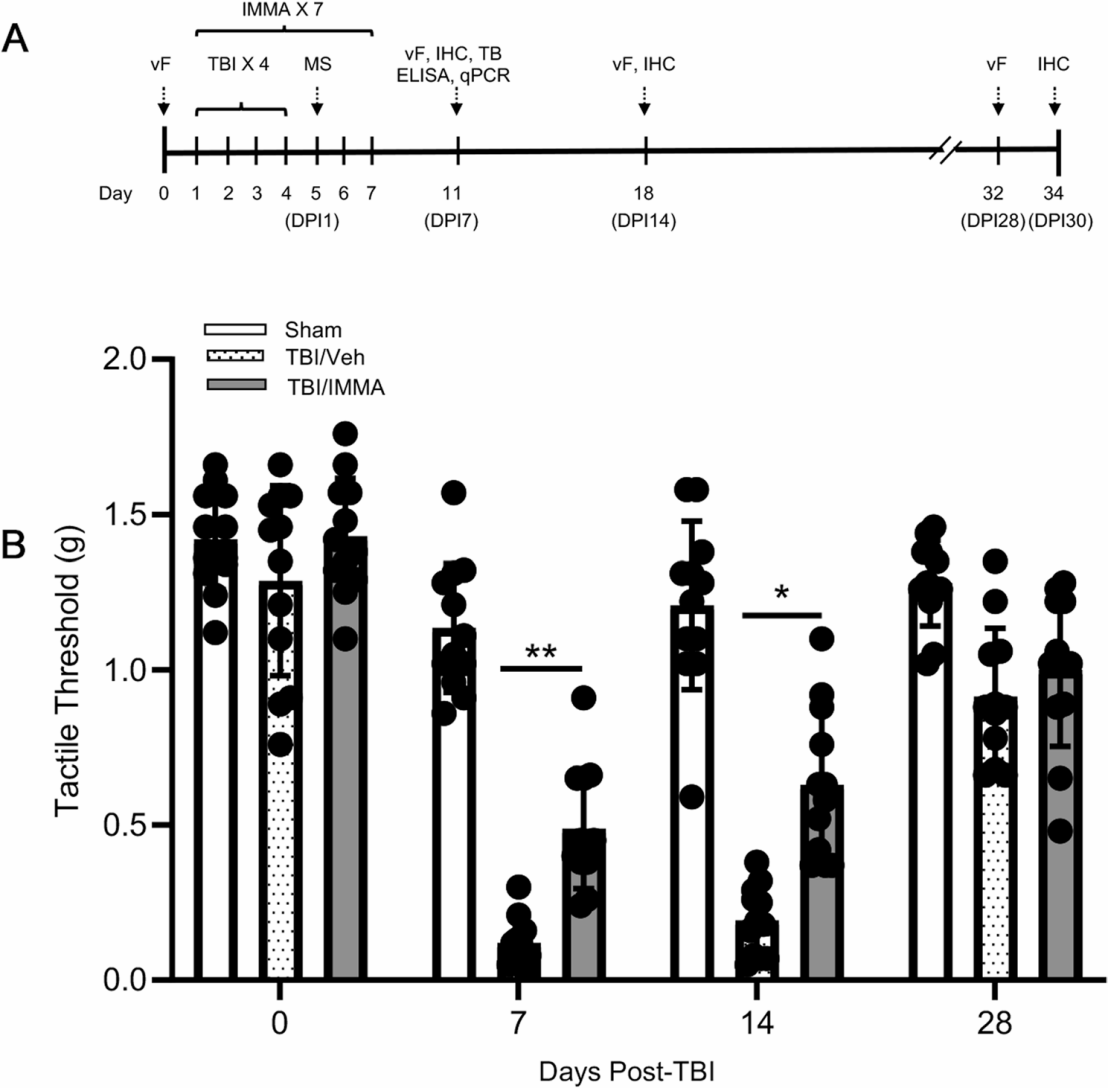
IMMA treatment suppressed periorbital allodynia in mice of repetitive mTBI. **A** Time line of experimental procedures. Mice received four repetitive mild TBI impacts (TBI ×4) and treated with IMMA for 7 consecutive days (IMMA ×7). Mass spectrometry (MS), von Frey test (vF), toluidine blue staining (TB), immunohistochemistry (IHC), PCR and enzyme linked immunosorbent assay (ELISA) were performed at the indicated days post-injury (DPI). **B** On days 0, 7, 14 and 28 post-TBI, mechanical periorbital tactile thresholds were evaluated by the “Up-Down” method. On days 7 and 14 post-TBI, compared to the sham group animals, the vehicle group showed a significantly reduced threshold. Animals treated with IMMA displayed significantly reduced mechanical response than that in the vehicle animals. *, *p* < 0.05, **, *p* < 0.01 were obtained when the TBI/vehicle group was compared to the IMMA treated group. At 28 days post-TBI, both TBI/vehicle and TBI/IMMA treatment groups displayed comparable periorbital thresholds to the sham group (mean ± SEM. *n* = 12)

To evaluate cephalic mechanical hypersensitivity, periorbital thresholds were assessed at baseline (day 0, prior to TBI), and at 7, 14, and 28 days post-injury using von Frey filaments as illustrated in Fig. 1A. A two-way repeated measures ANOVA on day 7 and day 14 revealed a significant main effect of treatment group (F (2,132) = 113.67, *p* < 0.0001) and time (F (3,132) = 113.99, *p* < 0.0001), as well as a significant group × time interaction (F (6,132) = 15.21, *p* = 0.0016). Post hoc comparisons showed that rmTBI/vehicle mice exhibited significantly reduced periorbital thresholds at both day 7 and day 14 compared to sham controls (*p* < 0.01). Mice treated with IMMA exhibited significantly higher pain thresholds compared to those receiving vehicle treatment at both time points (*p* < 0.05), indicating a reduction in allodynia (Fig. 1B). By day 28, periorbital thresholds in both rmTBI/vehicle and rmTBI/IMMA groups returned to levels comparable to the sham group (Fig. 1B). Baseline thresholds did not significantly differ among groups (F (2,23) = 1.49, *p* = 0.241), although the rmTBI mice had a trend toward slightly lower thresholds. This variation was not statistically significant and may reflect baseline variability rather than group-specific effects, as blinding was maintained throughout behavioral testing.

Time of righting reflex latency (RRL) after each TBI impact was measured as a loss of consciousness due to anesthesia and TBI. Sham animals were anesthetized with isoflurane for an equivalent duration without impact. rmTBI/vehicle mice exhibited significantly prolonged RRL compared to sham controls on days 1, 2, and 4 after the impact. rmTBI/IMMA mice also showed remarkably longer RRL than sham mice on all four days following injury. No differences in RRL were observed between the rmTBI/vehicle and rmTBI/IMMA groups at any time point. RRL values remained consistent across the four days within each experimental group (Supplementary Fig. 1).

### IMMA reduces microglia activation in the trigeminal nucleus caudalis

**Fig. 2:**
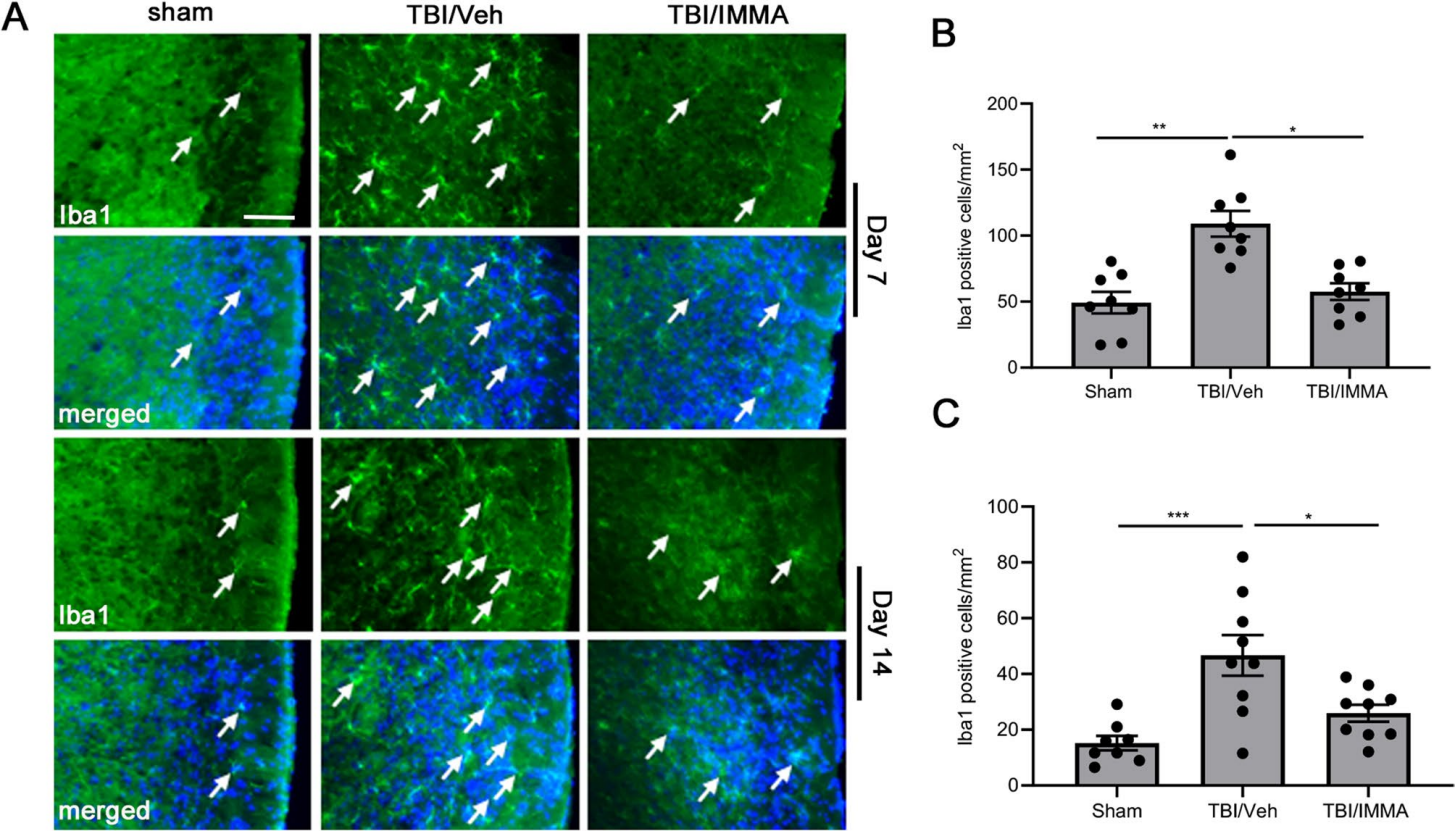
The increased microglia accumulation in the trigeminal nucleus caudalis (TNC) of the mTBI mice was attenuated by IMMA treatment. On days 7 and 14 post-TBI, the Iba1-positive macrophages/microglia immunostaining cells (indicated by arrows) increased in the TNC of the mTBI mice, that was reduced by IMMA treatment (**A**). The lower panels show co-staining with DAPI. Quantification showed that there was a 2-3 folds increase of the Iba1 positive cells in the TBI/vehicle group compared to the sham group in TNC at both 7 and 14 days post-TBI, and the increase was significantly attenuated by IMMA treatment (**B**, **C**). *, *p* < 0.05, **, *p* < 0.01 and ***p < 0.001 (mean ± SEM. *n* = 8/group). Scale bar = 50 μm

Microglia activation was assessed via immunohistochemistry using an anti-Iba1 antibody with staining performed on coronal TNC Sect. (20 μm) at 7 and 14 days post-TBI (Fig. 2A). Compared to the sham group, the rmTBI/vehicle group showed a significant increase in Iba1-immunopositive microglia/macrophages (137.1 ± 14.48 cells/mm² on day 7 (Fig. 2B), 46.67 ± 7.26 cells/mm² on day 14 (Fig. 2C) vs. 45.74 ± 4.54 cells/mm² on day 7, 15.19 ± 2.56 cells/mm² on day 14 in sham group, *p* < 0.001), suggesting an enhanced neuroinflammatory response post-TBI [29]. IMMA treatment substantially reduced Iba1 immunoreactivity (85.73 ± 7.17 cells/mm² on day 7 (*p* < 0.05 vs. vehicle, F (2,21) = 15.679. Figure 2B), and 26.92 ± 3.02 cells/mm² on day 14 (*p* < 0.05 vs. vehicle, F (2,27) = 15.981. Figure 2C). On day 30, there were no significant differences in the number of Iba1-positive cells among the experimental groups (6–14 cells/mm²; Supplementary Fig. 2).

### IMMA attenuates CGRP expression in the TNC

**Fig. 3:**
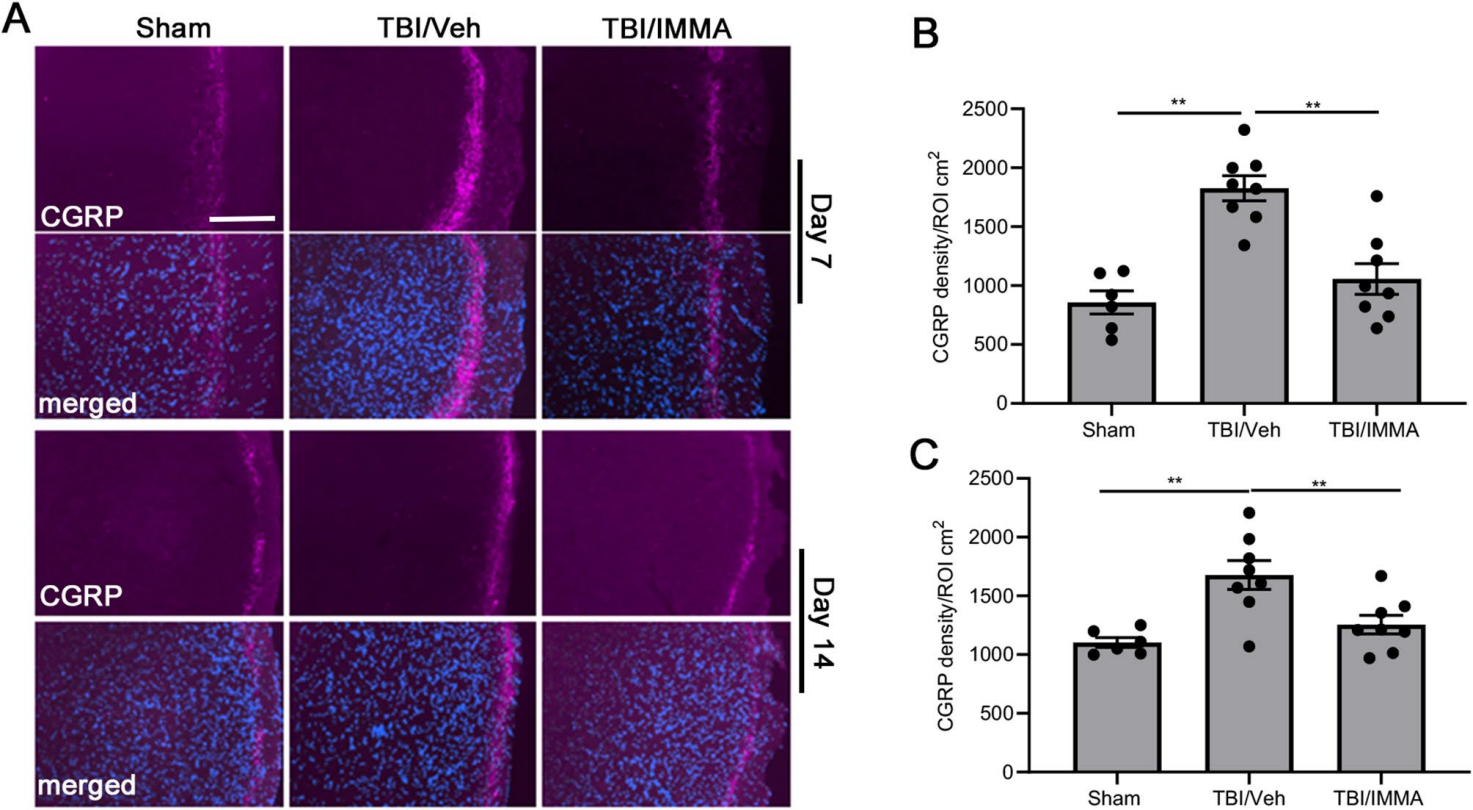
Treatment with IMMA inhibited the expression of CGRP in the TNC of mTBI mice. On days 7 and 14 post-TBI, there was increased CGRP-positive immunostaining in the TNC of the mTBI mice compared to the sham mice. Treatment with IMMA alleviated the CGRP positive immunostaining (**A**). The lower panels show co-staining with DAPI. The immunofluorescence intensity of CGRP in TNC was quantified with ImageJ software (**B**, **C**). The intensity of positive CGRP immunostaining in the TNC of mTBI mice was significantly higher than that in the sham group and greatly reduced by IMMA at 7 day (**B**) and 14 day (**C**) post-TBI. **, *p* < 0.01 were obtained when the IMMA treated group compared to the TBI/vehicle group (mean ± SEM. *n* = 10/group). Scale bar = 50 μm

CGRP immunoreactivity, assessed using an anti-CGRP antibody, was predominantly localized to the superficial laminae (I-II) of the TNC (Fig. 3A), a region implicated in trigeminal nociception [30]. At 7 days post-TBI, the rmTBI/vehicle group showed a 2-fold increase in CGRP-positive area (1837.73 ± 136.32/mm² vs. 841.77 ± 61.65/mm² in sham, ***, *p* < 0.001 (Fig. 3B), suggesting a heightened nociceptive response. IMMA treatment significantly lowered the expression of CGRP to 1149.51 ± 147.18/mm², F (2,27) = 17.713 (Fig. 3B). The expression of CGRP was also significantly increased at 14 days post-TBI and reduced by IMMA treatment, F (2,27) = 7.891 (Fig. 3C). At 30 days post-TBI, no significant differences were observed among the experimental groups (Supplementary Fig. 3). These results align with the CGRP’s role as a key mediator of PTH and migraine-like pain [44, 45].

### IMMA ameliorates astrocyte activation in the TNC

**Fig. 4:**
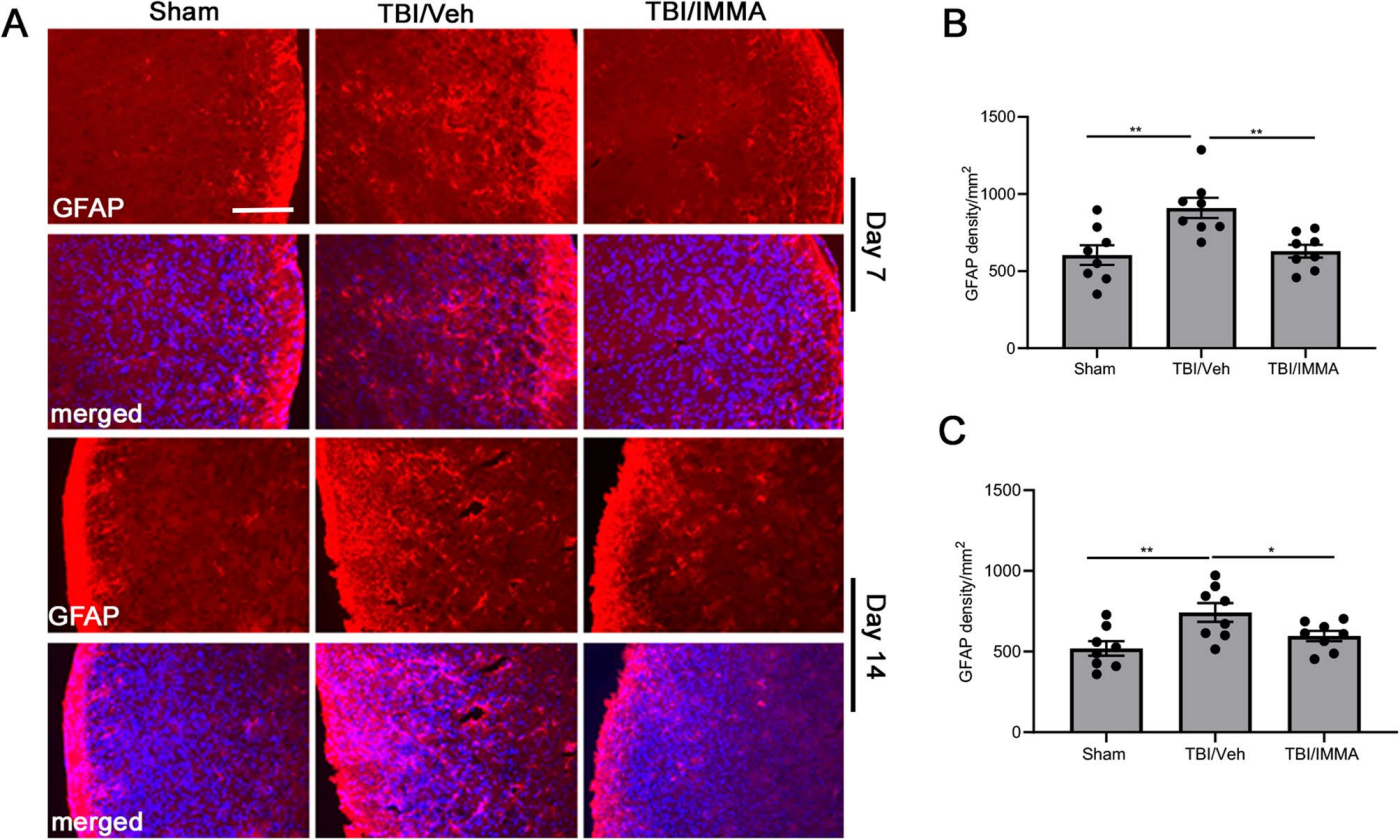
The activation of astrocytes in TNC of mTBI mice was suppressed by IMMA administration. On days 7 and 14 post-TBI, the GFAP-positive immunostaining was increased in the TNC of the mTBI mice and reduced in the IMMA treatment group (**A**). Quantitative analysis indicated that the TBI/vehicle group animals had significantly enhanced GFAP positive immunostaining in the TNC region on days 7 and 14 post-TBI compared to the sham group (**, *p* < 0.01). The TBI animals that received IMMA treatment had remarkably attenuated GFAP immunostaining in the TNC at 7 (**, *p* < 0.01 compared to the TBI/vehicle group) (**B**) and 14 days post-TBI (*, *p* < 0.05 compared to the TBI/vehicle group) (**C**) (mean ± SEM. *n* = 8/group). Scale bar = 50 μm

Astrocyte activation was evaluated using an anti-GFAP antibody (Sigma-Aldrich, 1:500) in TNC tissues at 7, and 14 days post-TBI (Fig. 4A). The rmTBI/vehicle group displayed robust GFAP immunoreactivity (878.88 ± 31.69/mm²) compared to sham (590 ± 50.28/mm², *p* < 0.01), indicative of reactive gliosis post-TBI. IMMA treatment significantly reduced GFAP immunoreactivity to 664 ± 30.54/mm² on day 7 (*p* < 0.01 vs. vehicle, F (2.21) = 8.521) (Fig. 4B). On day 14 post-TBI, the GFAP expression was also significantly increased in the TBI/vehicle group and reduced in the IMMA treatment group F (2,21) = 6.045 (Fig. 4C). Although the expression of GFAP seemed to be greater in the TBI/vehicle group at 30 days, no significant difference was observed when it was compared to the sham group or the IMMA treatment group (Supplementary Fig. 4).

### IMMA reduces CGRP and Iba1 expression in the trigeminal ganglion

**Fig. 5:**
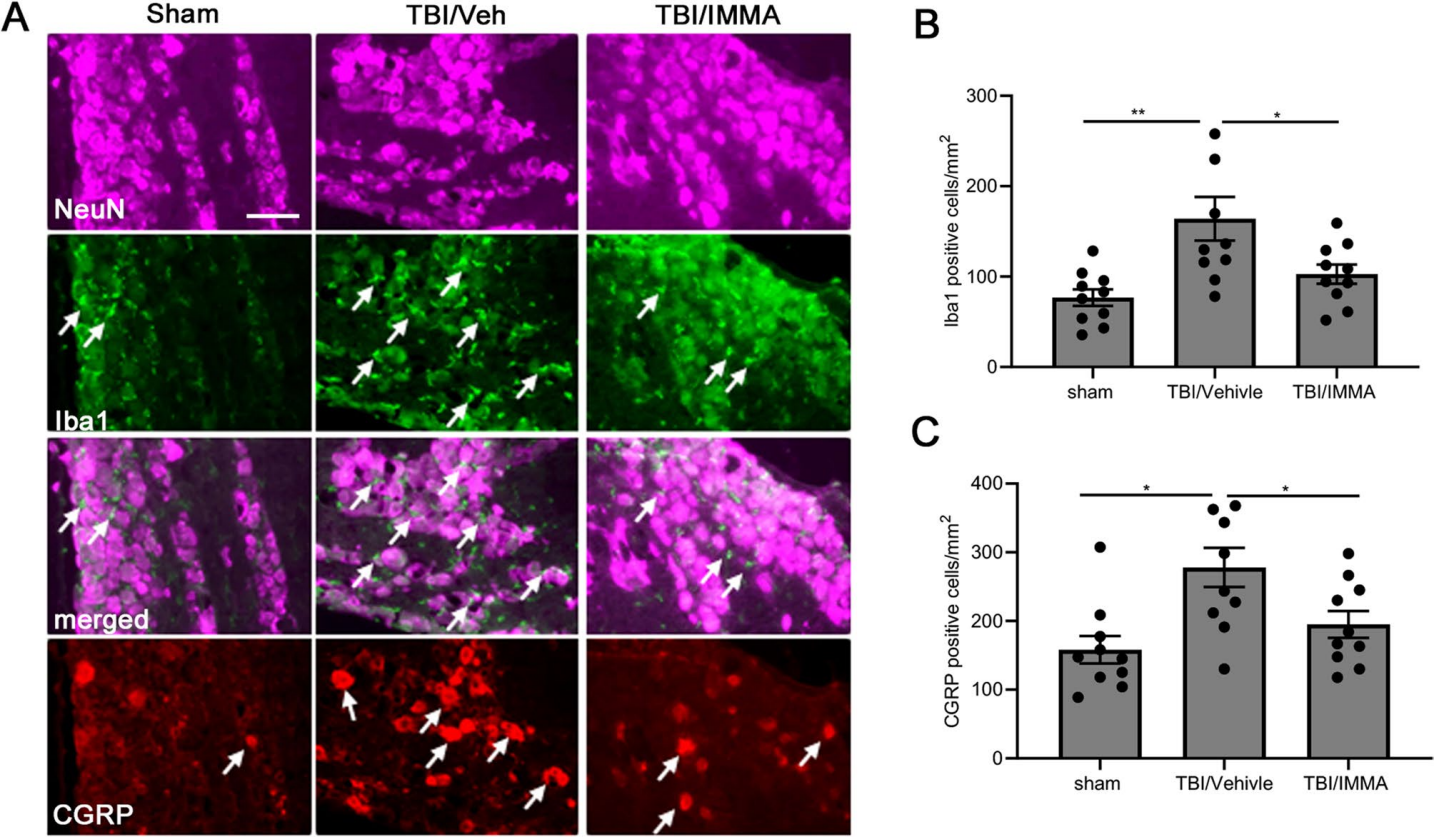
IMMA treatment attenuated the increased expression of Iba1 and CGRP in the TBI mouse TG. On day 7 post-TBI, the CGRP production and microglia infiltration (indicated by arrows) were examined with anti-CGRP and anti-Iba1 antibodies in the TG tissues. Compared to the sham group and IMMA treated TBI group, the number of CGRP and Iba1 positive cells in the TG of TBI/vehicle group was found obviously increased (**A**). IMMA treatment significantly reduced Iba1 positive cells compared to that in the TBI/vehicle group (**B**). The CGRP immunopositive cells in the TBI/vehicle group were also significantly attenuated with the IMMA treatment (**C**). *p < 0.05, and **p < 0.01 (mean ± SEM. *n* = 10/group). Scale bar = 50 μm

TG sections were stained with anti-Iba1 and anti-CGRP antibodies at 7 days post-TBI (Fig. 5A). The expression of Iba1 positive macrophages was dramatically increased in the rmTBI/vehicle group and reduced in the IMMA treatment group (Fig. 5A). The Iba1 expressing macrophages were increased from 45.46 ± 6.56 cells/mm² in the sham group to 143.8 ± 21.65 cells/mm² in the TBI/vehicle group (*p* < 0.01), and treatment with IMMA reduced the expression of macrophages to 77.61 ± 7.05 cells/mm² (*p* < 0.05 vs. vehicle), F (2,27) = 20.079 (Fig. 5B). The rmTBI/vehicle group also showed a 2.2-fold increase in CGRP-positive cells (276.39 ± 54.91 vs. 134.94 ± 17.08 in sham, *p* < 0.05) (Fig. 5C), which was significantly reduced in the IMMA treatment group (*p* < 0.05, Fig. 5C), F (2,27) = 7.012. These results demonstrated that IMMA could suppress macrophage infiltration and CGRP release, key contributors to trigeminal pain in PTH.

### IMMA reduces meningeal mast cell degranulation

**Fig. 6:**
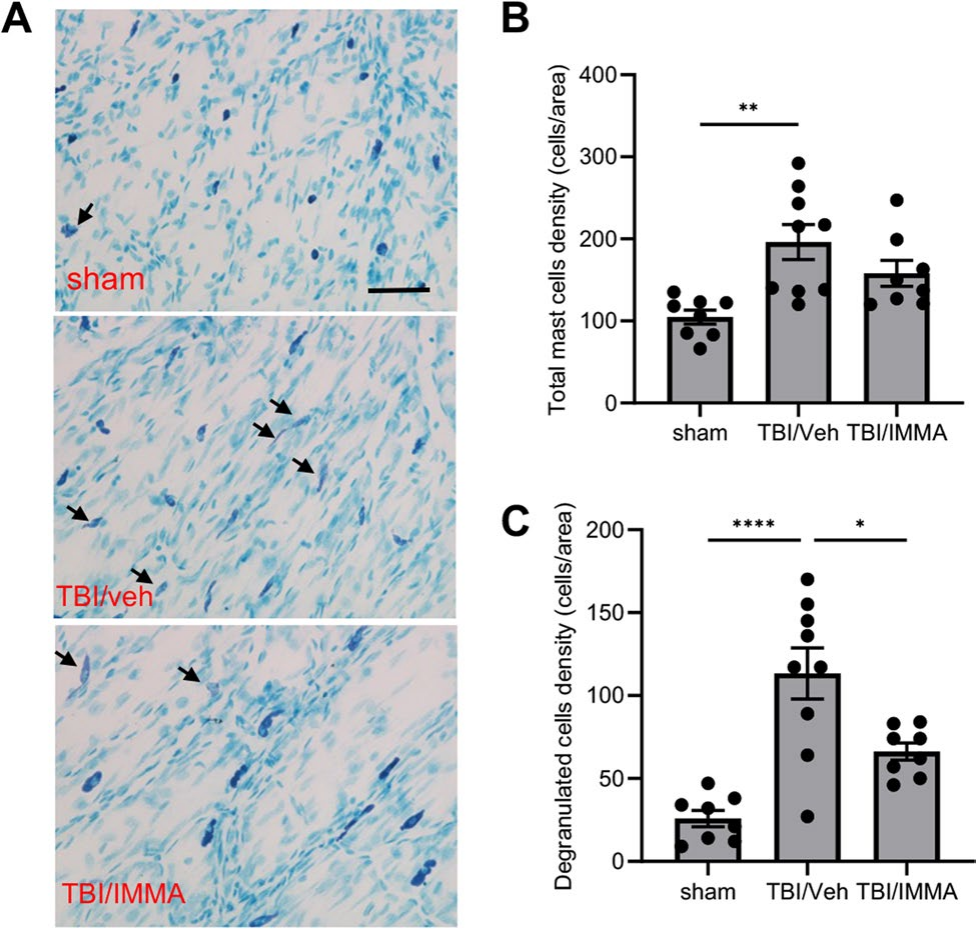
Treatment with IMMA reduced mast cell degranulation in the TBI mouse meninges. At 7 days post-TBI, meninges were dissected and stained with toluidine blue. Degranulated mast cells identified by faint staining due to extensive granule secretion were indicated by arrows (**A**). The number of total mast cells and degranulated mast cells were counted. Both the total mast cell and the degranulated mast cell densities were significantly increased in the TBI animals but reduced by IMMA treatment (**B**, **C**). *p < 0.05, **p < 0.01 and ****p < 0.0001 (mean ± SEM. *n* = 8/group). Scale bar = 100 μm

At 7 days post-TBI, meninges were dissected from cranial bones, mounted on slides, and stained with toluidine blue. Normal mast cells were densely stained due to abundant intracellular granules, while the degranulated mast cells were indicated by faint staining due to extensive granule secretion (Fig. 6A). The rmTBI/vehicle group showed a significant increase in total mast cells (196 ± 21 cells/area), while mast cell density in sham group was 105 ± 8.5 cells/area (*p* < 0.01). And 20% of total mast cells were reduced by IMMA treatment (158 ± 16 cells/area, F (2,22) = 7.695) (Fig. 6B). Degranulated mast cell density was increased by more than 4-fold in the rmTBI/vehicle group (113 ± 15 cells/area) compared to the sham group (26 ± 4.9 cells/area). IMMA treatment significantly reduced the degranulated cells (66 ± 5.2 cells/area. *p* < 0.05, F (2,22) = 18.10) (Fig. 6C).

### IMMA elevates brain AEA levels

**Fig. 7:**
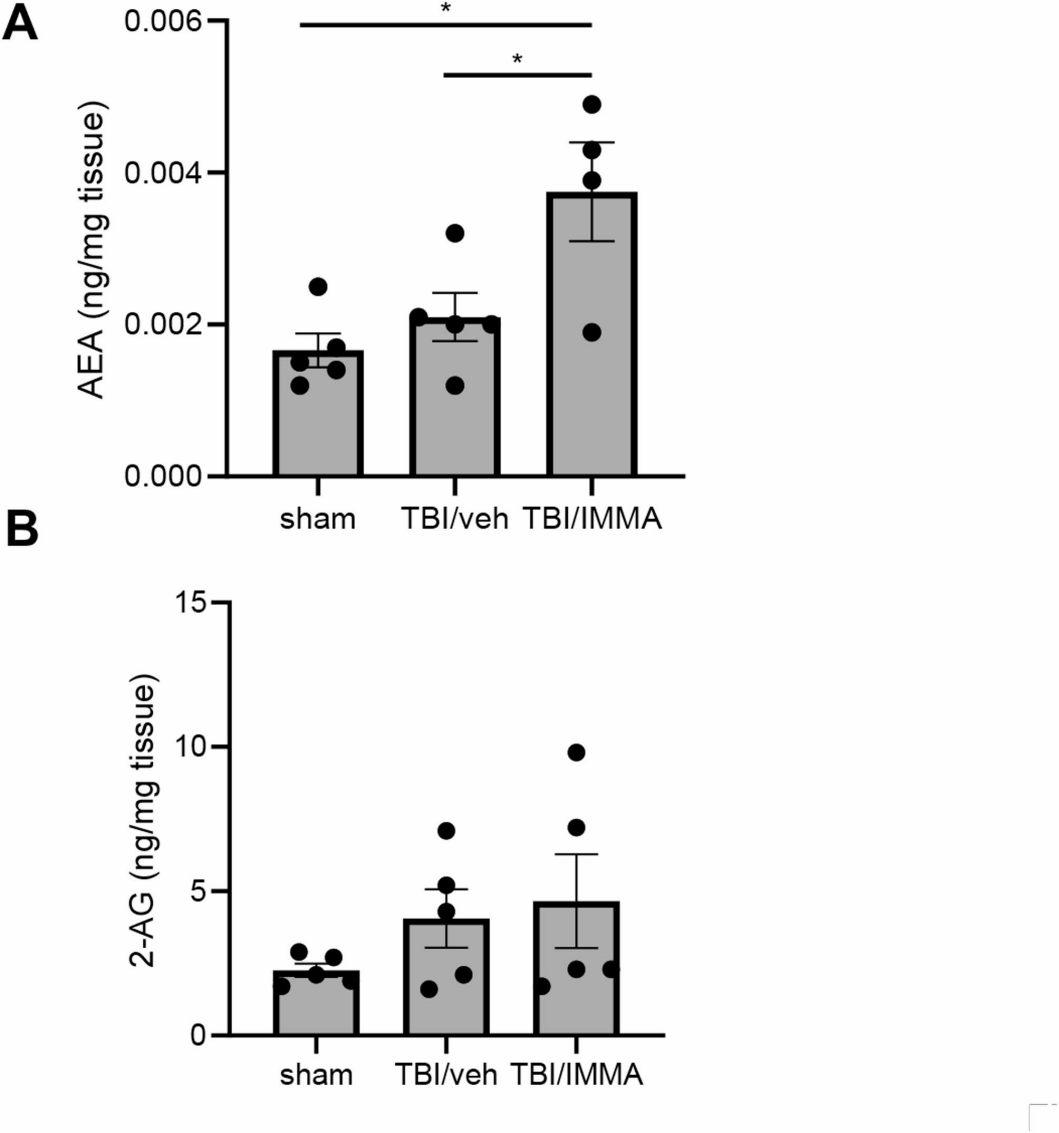
IMMA treatment significantly increased AEA, but not 2-AG in the TBI mice. At 1 day post-TBI, the levels of the AEA and 2-AG in cerebral cortex were quantified with LC-MS/MS. TBI/vehicle group showed slightly increased AEA compared to the sham. The AEA levels in the IMMA treatment group were significantly increased than that in the TBI/vehicle group. 2-AG levels in both TBI/vehicle and IMMA treated mice were increased compared to the sham animal, but there were no significant differences between the TBI/vehicle and TBI/IMMA treatment groups. *p < 0.05 (mean ± SEM. *n* = 5/group)

To determine the levels of endocannabinoids in TBI and drug treated groups, TBI mice were euthanized at 1 day post-TBI, and the AEA and 2-AG levels in the cerebral cortex were quantified by LC-MS/MS. No significant differences in AEA levels were observed between the TBI/vehicle group (2.1 ± 0.3 pg/mg brain tissue) and the sham group (1.7 ± 0.2 pg/mg, *p* > 0.05) (Fig. 7A). However, the rmTBI/IMMA group exhibited significantly elevated AEA (3.8 ± 0.7 pg/mg, *p* < 0.05 vs. vehicle and sham, F (2,11 = 7.093). For 2-AG, there was a non-significant increase in the rmTBI/vehicle (4.1 ± 1.0 ng/mg) compared to the sham group (2.3 ± 0.2 ng/mg, *p* > 0.05), and the IMMA treatment group (4.7 ± 1.6 ng/mg) was comparable to the rmTBI/vehicle group, (F (2,120 = 1.259). This result aligns with previous findings that IMMA treatment significantly increased brain levels of AEA, though to a lesser extent for 2-AG [28].

### IMMA does not alter PGE_2_ production in the trigeminal ganglion

**Fig. 8:**
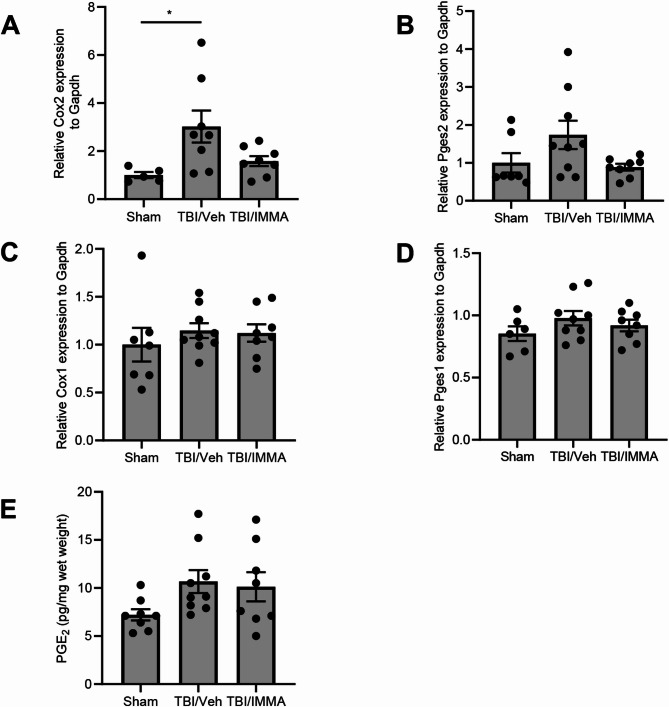
IMMA treatment did not affect the expression of COXs, PGESs and the production of PGE_2_ in the TBI mouse TG. At 7 days post-TBI, gene expression of PGE_2_ biosynthetic enzymes, Cox-1, Cox-2, Pges1 and Pges2 was quantified by qPCR. The Cox-2 expression was increased in the TBI/vehicle group compared to the sham (*p* < 0.05) but reduced by IMMA treatment (**A**). The increased expression of Pges2 was also observed in the TBI/vehicle group compared to the sham, but reduced by IMMA treatment (**B**). The expression of Cox-1 (**C**) and Pges1 (**D**) was not changed among the groups. PGE_2_ levels in TG measured by ELISA showed increase in the TBI/vehicle and TBI/IMMA groups compared to sham group (**E**). (mean ± SEM. *n* = 8–9/group)

To evaluate gene regulation involved in PGE₂ biosynthesis, expression levels of COX-1, COX-2, prostaglandin E synthase-1 (Pges1), and Pges2 were quantified by qRT-PCR in TG at 7 days post-TBI. Compared to the sham group, the rmTBI/vehicle group exhibited a 2.8-fold increase in COX-2 expression (Fig. 8A; F (2,18) = 4.809) and a 1.7-fold increase in Pges2 expression (Fig. 8B; F (2,21) = 2.882). IMMA treatment reduced their expression levels, although there was no significant difference between the rmTBI/vehicle and the rmTBI/IMMA groups. Conversely, the expression levels of COX-1 (Fig. 8C) and Pges1 (Fig. 8D) were the same among the experimental groups (F (2,21) = 0.4437 and F (2,20) = 1.203, respectively). PGE2 production was quantified using ELISA (Fig. 8E). The PGE_2_ levels were increased by approximately 50% in the rmTBI/vehicle (10.7 ± 1.2 pg/mg) compared to the sham group (7.2 ± 0.6 pg/mg). IMMA treated group showed comparable levels to the TBI/vehicle group (10.1 ± 1.5 pg/mg; F (2,22) = 2.509).

## Discussion

This study demonstrates that IMMA, a substrate-selective COX-2 inhibitor, effectively mitigates PTH in a repetitive mTBI mouse model by reducing periorbital allodynia, enhancing endocannabinoid signaling, and suppressing neuroinflammation without altering PGE_2_ production. Unlike the traditional COX-2 inhibitors, IMMA preserves physiological prostaglandin synthesis while selectively inhibiting COX-2-mediated endocannabinoid degradation. Thus, IMMA represents a mechanistically distinct and safer alternative to traditional anti-inflammatory therapies. These findings position IMMA as a novel therapeutic candidate for PTH.

### IMMA preserves endocannabinoid signaling through COX-2 substrate selectivity

The therapeutic effect of IMMA likely stems from its selective inhibition of COX-2-mediated endocannabinoid metabolism, a process that preserves AEA and 2-AG levels while avoiding the inhibition of AA metabolism seen with celecoxib and other conventional COX-2 inhibitors [27]. COX-2 plays a pivotal role in oxygenating endocannabinoids into pro-inflammatory PG-EAs and PG-Gs, which reduce the bioavailability of AEA and 2-AG, and exacerbate inflammation and nociception [23]. Given the upregulation of COX-2 in TG following TBI, it is likely that the increased oxygenation of endocannabinoid contributes to the PTH pathogenesis.

Our findings that PGE_2_ levels in the TG remain unchanged following IMMA treatment further reinforce its substrate selectivity. Unlike the broad-spectrum COX-2 inhibitors that indiscriminately suppress PGE_2_ synthesis and endocannabinoid oxygenation, IMMA selectively inhibits the latter while preserving the physiological prostaglandins, ensuring the maintenance of essential prostaglandin homeostatic functions [27]. Since PGE_2_ also plays a protective role in vascular function and gastrointestinal integrity, its suppression by the traditional NSAIDs may result in the cardiovascular and gastrointestinal toxicity [46]. By preserving PGE_2_ while selectively inhibiting COX-2 mediated endocannabinoid oxygenation, IMMA enhances endogenous pain modulation without inducing the systemic risks associated with NSAIDs or the adverse effects linked to cannabinoid-based therapies [28].

Interestingly, although IMMA significantly elevated cortical AEA levels, repetitive mTBI did not produce a measurable reduction in AEA compared to sham controls. This suggests that IMMA’s therapeutic benefit may not rely solely on restoring deficient levels of AEA, but rather on amplifying endocannabinoid tone beyond baseline to suppress neuroinflammatory signaling. It is also possible that localized or cell-specific disruptions in AEA signaling occurred but were undetectable in bulk cortical measurements. Prior studies have shown that AEA function can be impaired through CB1 receptor desensitization or altered enzyme activity, even when tissue concentrations remain unchanged [47]. Further investigation is needed to determine whether IMMA enhances AEA bioavailability at synaptic or subcellular levels relevant to pain modulation and glial reactivity.

### IMMA suppresses glial activation and CGRP expression in the trigeminal circuits

Neuroinflammation, driven by glial activation and CGRP upregulation, is a major contributor to the pathogenesis of PTH and migraine [48–50]. TBI-induced microglial and astrocyte activation triggers the release of pro-inflammatory cytokines such as TNF-α and IL-1β, which amplify trigeminal sensitization and prolong headache-like pain [51]. CGRP, a potent vasodilator and nociceptive mediator elevated in the TNC and meninges during PTH, can further trigger the release of inflammatory mediators and contribute to the development of headache pain [52]. IMMA treatment significantly reduced the expression of microglia and astrocytes in the TNC, supporting its potent anti-inflammatory effects. The reduction in CGRP expression by IMMA also mirrors previous findings that underscore CGRP’s pivotal role in trigeminal nociception across various headache disorders [52].

Unlike the CGRP receptor antagonists, IMMA is likely to reduce the CGRP synthesis by modulating COX-2-endocannabinoid interaction. COX-2 upregulation following TBI promotes CGRP expression by driving prostaglandin synthesis and AEA degradation [23, 53]. By preserving AEA levels, IMMA enhances the CB1 receptor activation, and suppresses the CGRP synthesis in the trigeminal system [54]. Our finding that IMMA significantly reduces CGRP expression in the TNC is in harmony with this potential mechanism of action.

### IMMA stabilizes meningeal mast cells and reduces peripheral inflammation

Meningeal mast cells are key mediators of neurogenic inflammation, releasing histamine, serotonin, and proteases that sensitize meningeal nociceptors and exacerbate trigeminal pain [55]. TBI-induced mast cell degranulation disrupts meningeal homeostasis and worsens periorbital allodynia [56].

A previous study using a weight-drop TBI rat model found a 60% mast cell degranulation accompanied with the release of nociceptive mediators, such as histamine and proteases [57]. Our current study showed that both the total and degranulated meninge mast cells were significantly increased in TBI animals and reduced by IMMA treatment. The inhibitory effect of IMMA is likely mediated through CB2 receptor activation, given that AEA suppresses FcεR1 mediated degranulation and cytokine production from mast cells via CB2-GPR55 heteromer signaling [58]. By preventing COX-2-mediated conversion of AEA into PG-EAs, IMMA enhances the CB2-mediated mast cell inhibition, reducing inflammatory mediator release and dampening meningeal inflammation.

### IMMA outperforms conventional treatments for PTH

Current treatments for PTH, such as NSAIDs, triptans, and CGRP antagonists, have significantly advanced clinical management and mechanistic understanding of headache disorders. Treatment with NSAIDs provides relief in fewer than 50% of cases [59, 60], but can also cause gastrointestinal and cardiovascular side effects [46]. In addition to NSAIDs, there are several other options for migraine treatments including 5-HT receptor agonists, such as triptans or ergotamine [61], CGRP inhibition such as gepant or erenumab [7, 10], anticonvulsants, antihypertensives and antidepressants [62–64]. However, these treatments are less effective to ameliorate chronic symptoms and are contraindicated in patients with cardiovascular comorbidities [1, 2].

In comparison with the above-mentioned therapies, modulation of the endocannabinoid system may exert broader effects by targeting multifaceted pain pathways [17–19]. Preclinical studies have shown that augmentation of the endocannabinoid tone via inhibition of FAAH or MAGL can reduce inflammatory and neuropathic pain by increasing the levels of AEA or 2-AG [21, 65, 66]. However, chronic and complete inhibition of these enzymes may lead to CB1 receptor desensitization and unwanted side effects [67]. Thus, fine tuning of the endocannabinoid tone is crucial to achieve better therapeutic efficacy.

Our findings highlight the need for balanced modulation of the endocannabinoid system and support the exploration of alternative approaches like the substrate selective COX-2 inhibitors. These agents can maintain endocannabinoid signaling while minimizing the adverse effects associated with prolonged FAAH or MAGL inhibition. Therefore, the SSCIs, such as IMMA, may control the endocannabinoid system in a more targeted and physiologically sustainable manner to support the treatment of PTH.

Consistent with our previous studies [4], we found the significantly increased periorbital allodynia within 14 days post-injury, and at 30 days post-TBI, the cephalic tactile threshold, the accumulation of glial cells and the expression of CGRP in TNC returned to the sham controls. While IMMA has demonstrated efficacy in mitigating the acute phase of PTH, its ability to prevent the progression to persistent PTH following environmental triggers remains to be elucidated. Given the elevated prevalence of PTH and migraine in female populations [68–70], and the documented sexual dimorphism in endocannabinoid signaling within the trigeminal system and pain associated brain regions [71, 72], future studies employing female TBI animal models are needed to evaluate the therapeutic potential of IMMA in both acute and persistent phases of PTH.

## Conclusion

This study demonstrates that IMMA, a substrate-selective COX-2 inhibitor, alleviates acute PTH symptoms by reducing neuroinflammation, maintaining endocannabinoid signaling, and avoiding disruption of prostaglandin synthesis. By targeting multiple inflammatory pathways while preserving physiological homeostasis, IMMA offers a mechanistically distinct and promising therapeutic approach for acute PTH. However, as the study was limited to early post-injury timepoints and cortical endocannabinoid measurements, further investigation is needed to assess its efficacy in chronic PTH and in anatomically localized regions critical to headache pathophysiology. These future studies will be essential to establish IMMA’s full translational potential.

## Supplementary Information

Supplementary Material 1.

## Data Availability

Data are available from the corresponding author upon a reasonable request.

## Abbreviations

AA: Arachidonic acid
AEA: Anandamide
2-AG: 2-arachidonoyl glycerol
CB1R: Cannabinoid type 1 receptor
CB2R: Cannabinoid type 2 receptor
CGRP: Calcitonin gene-related peptide
CHIMERA: Closed head impact model of engineered rotational acceleration
COX-2: Cyclooxygenase-2
ECS: Endocannabinoid system
ELISA: Enzyme linked immunosorbent assay
FAAH: Fatty acid amide hydrolase
IHC: Immunohistochemistry
IMMA: Indomethacin morpholinamide
LC/MS: Liquid chromatography/mass spectrometry
MAGL: Monoacylglycerol lipase
mTBI: Mild traumatic brain injury
NSAIDs: Non-steroidal anti-inflammatory drugs
PG: Prostaglandins
PGE2: Prostaglandins E2
PG-EAs: Prostaglandin ethanolamides
PG-Gs: Prostaglandin glycerol esters
PTH: Posttraumatic headache
rmTBI: Repetitive mild traumatic brain injury
SSCIs: Substrate-selective COX-2 inhibitors
TBI: Traumatic brain injury
TG: Trigeminal ganglion
TNC: Trigeminal nucleus caudalis
