# Supplementary material for: Substrate-selective COX-2 inhibition by IMMA attenuates posttraumatic headache via endocannabinoid modulation and neuroinflammatory suppression
Source: J Headache Pain. 2025 Aug 12;26(1):183. doi: 10.1186/s10194-025-02116-x (PMC12341237; doi:10.1186/s10194-025-02116-x)
Supplement: Supplementary file 1 — Supplementary Material 1. [file 10194_2025_2116_MOESM1_ESM.pdf]

Supplementary Files:

Supplementary Figure 1. Repeated mTBI prolonged the righting reflex latency (RRL) compared to the sham controls.

RRL was measured immediately following each TBI impact. Sham mice were exposed to anesthesia for the same duration as TBI mice but did not receive an impact. RRL of the rmTBI/vehicle mice in three out of four TBIs and RRL of rmTBI/IMMA mice in all four TBIs, were significantly longer than that of sham mice, which is analogous to the loss of consciousness in human TBI. # $p<0.05$, ## $p<0.01$ and ### $p<0.001$ were obtained between the rmTBI/IMMA and sham. * $p<0.05$ and **$p<0.01$ represent statistical differences between the rmTBI/vehicle and the sham group. (mean ± SEM. n=12/group).

Supplementary Figure 2. No significant differences in microglial accumulation were observed in the TNC among experimental groups at 30 days post-TBI.

At 30 days post injury, Iba1-positive microglia/macrophage immunostaining in the TNC slightly increased in the TBI/vehicle group compared to that observed in the sham and IMMA-treated groups (A). Quantitative analysis confirmed no significant differences in microglial accumulation among the groups (B). Data are presented as mean ± SEM (n = 6 per group). Scale bar = 50 μm.

Supplementary Figure 3. CGRP expression in the TNC remained unchanged among groups at 30 days post-TBI.

CGRP-positive immunostaining in the TNC at 30 days post-mTBI showed a slight, but not remarkable increase in the TBI/vehicle group compared to the sham group (A). IMMA treatment did not reduce CGRP expression. Quantitative analysis of CGRP immunofluorescence intensity

revealed no significant differences among the experimental groups (B). Data are presented as mean ± SEM (n = 8 per group). Scale bar = 50 μm.

Supplementary Figure 4. Astrocyte activation in the TNC did not differ significantly across groups at 30 days post-TBI.

GFAP-positive immunostaining in the TNC was modestly elevated in the TBI/vehicle group compared to the sham and IMMA-treated groups at 30 days post-injury (A). However, quantitative analysis indicated that these differences were not statistically significant (B). Data are presented as mean ± SEM (n = 6 per group). Scale bar = 50 μm.

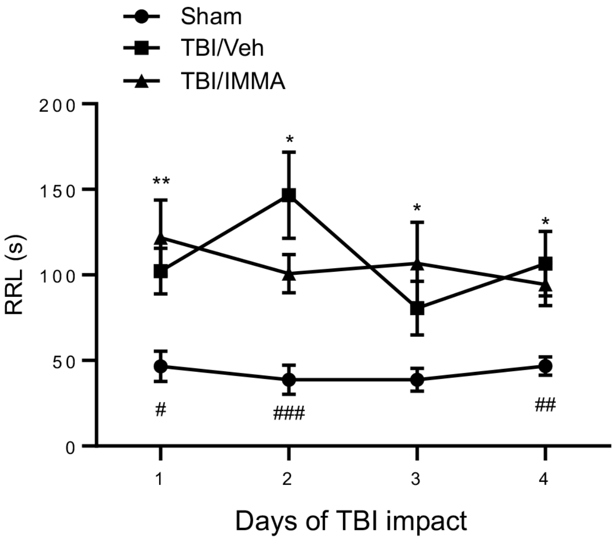

Supplementary figure 1

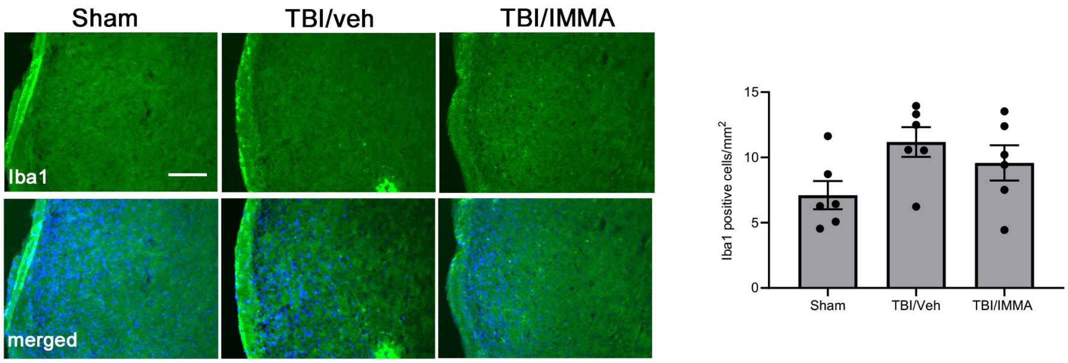

Supplementary figure 2

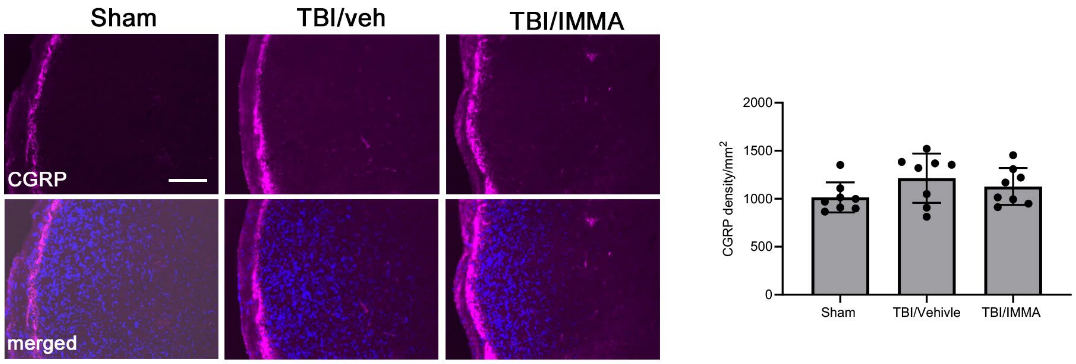

Supplementary figure 3

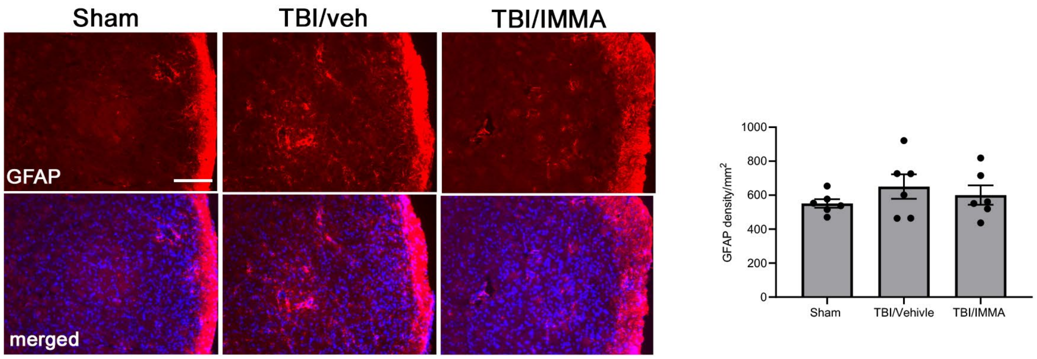

Supplementary figure 4
